# A rare case of urethral metastasis from colonic origin

**DOI:** 10.1016/j.eucr.2026.103412

**Published:** 2026-03-14

**Authors:** Faiyaz Rahman, Shravankrishna Ananthapadmanabhan, Jeremy Saad, Bishoy Hanna, Isaac Thangasamy, Celalettin Varol

**Affiliations:** a Nepean Urology Research Group, Nepean Hospital, Kingswood, New South Wales, Australia; b University of Sydney, Faculty of Medicine, Sydney, New South Wales, Australia

**Keywords:** Metastatic colorectal adenocarcinoma, Urethral tumour, Cystoscopy

## Abstract

Secondary urethral tumours are rare, and metastasis from colorectal cancer is limited to a few case reports. Management is challenging given its rarity. We report the case of a 78-year-old male with colorectal adenocarcinoma presenting with lower urinary tract symptoms, with urethral metastasis identified during cystoscopy. Local clearance was achieved by cold cup biopsy supplemented by a multidisciplinary approach to overall patient care. This case highlights the need for thorough evaluation of new urinary symptoms in patients with a history of cancer, and the importance of multidisciplinary teams in managing rare cancers.

## 1 Introduction

Urethral tumours are a rare occurrence, accounting for less than 1% of all cancers affecting the urinary tract.^1^ Even more infrequent are secondary malignancies that affect the urethra, with the majority originating from primary sites within the genitourinary system.^2^ Previous literature has proposed various mechanisms including direct extension, lymphatic or venous spread.^3^

Due to the scarcity of such cases, there are limited reports in the surgical literature documenting instances of rectal adenocarcinoma metastasizing to the urethra (Table 1).

**Table 1:** Case reports of urethral metastatic disease with primary colorectal malignancy post-2000.

Author and Year	Primary Site	Urethral Site	Management of urethral lesion	Outcome (if specified)
Chitale, Burgess, Sethia et al., 2004^4^	Sigmoid colon	External urethral meatus	Radical cystourethrectomy with bilateral salpingo-oophorectomy and urinary diversion	No recurrence at 30 months post-op.
Chitale, Burgess, Sethia et al., 2004^4^	Rectum	Bulbar urethra	Localised resection	Deceased at 6 months post-biopsy
Chang, Chuang, Ng et al., 2007^5^	Ascending colon	Penile urethra	Partial penectomy + right inguinal regional lymph node dissection + radiotherapy + adjuvant chemotherapy	No recurrence at 20 months
Noorani, Rao, Callaghan. 2007^3^	Sigmoid colon	Urethral meatus	Neoadjuvant chemotherapy with plan for anterior resection and pelvic exenteration.	Unspecified.
Seo, Kim, Kim et al., 2011^6^	Sigmoid Colon	Unspecified	Localised resection + adjuvant chemotherapy	Deceased at time of publication
Chang, Hsu, Chien et al., 2014^7^	Rectum	Penile urethra	Localised resection + adjuvant chemotherapy	No recurrence at 12 months
Karakose, Aydogdu, Atesci. 2014^8^	Sigmoid colon	Penile + bulbar urethra	Localised resection + adjuvant chemotherapy	Unspecified
Kazama, Kitayama, Sunami et al., 2014^9^	Sigmoid colon	Bulbar urethra	Localised resection + adjuvant chemotherapy	No recurrence at 34 months
Yagihashi, Arakaki. 2015^10^	Sigmoid colon	Bulbar urethra	Total urethrectomy	No recurrence at 15 months
Benatiya, El Asri, Rais. 2018^11^	Sigmoid colon/rectum	Urethral meatus	Nil intervention	Deceased at 1 month post-diagnosis

In this report, we present a unique case in which a patient presented with lower urinary tract symptoms and was subsequently diagnosed with urethral metastasis originating from rectal adenocarcinoma. This case underscores the importance of considering uncommon metastatic pathways when evaluating patients with genitourinary symptoms and highlights the need for a thorough diagnostic approach in such instances.

### 1.1 Case description

A 78-year-old male patient was referred to a urologist due to the need for investigation and management of urinary retention. He initially presented to the emergency department in urinary retention and would have a catheter inserted. The patient's urological history included a previous laser prostatectomy performed for the management of benign prostatic hyperplasia. Other relevant background history includes rectal adenocarcinoma, which had been managed with a low anterior resection followed by adjuvant capecitabine therapy. This malignancy had progressed to metastatic disease, involving both the liver and lungs, leading to the initiation of palliative FOLFOX treatment.

On review, he reported a history of voiding lower urinary tract symptoms, gradually worsening over a 12-month period. Initial investigations included a renal tract ultrasound which demonstrated a pre-void volume of 696ml with a significant post-void residual volume of 478mL and a prostate volume of 27mL. Uroflowmetry revealed a reduced maximal flow rate of 2mL/sec, suggestive of bladder outlet obstruction.

**Fig. 1:**
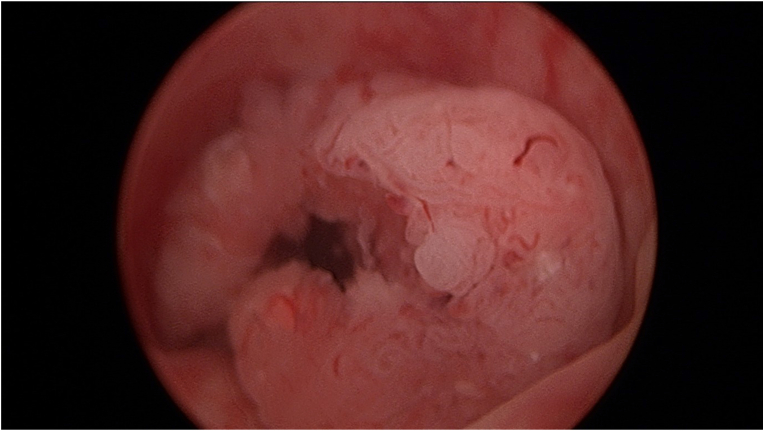
– Papillary tumour occluding the penile urethra on cystoscopy.

The patient then underwent a cystoscopy, which revealed a papillary tumour occluding the entire penile urethra (Fig. 1). The prostate was non-enlarged, and a small capacity bladder with no trabeculations was seen. A biopsy was taken of the mass and a 20Fr urethral catheter was placed at the time of cystoscopy after dilatation over a guidewire. The lesion was cleared using cold cup biopsy forceps to form an open channel and aid urinary flow. Transurethral tumour resection at the time of cystoscopy was not performed. A subsequent trial of void was unsuccessful, and a suprapubic catheter was then placed for long-term management.

Histopathologic examination of the papillary lesion showed moderately differentiated adenocarcinoma and immunohistochemical stains were positive for CDX2 and CK20 and negative for CK7, in keeping with a metastatic lesion of the urethra from colorectal origin. The case was discussed at a multidisciplinary meeting, and a consensus was reached to continue palliative chemotherapy with interval CT scans to monitor disease progression. With further progression of metastatic deposits, a decision was made to transition to supportive end-of-life measures.

## 2 Discussion

Urethral tumours are a rare occurrence within the spectrum of urological malignancies, constituting less than 1% of all cases and exhibiting a male predilection with a 3 to 1 ratio. These tumours are predominantly primary masses, with secondary urethral tumours being exceptionally rare, often documented as isolated case reports. When secondary urethral tumours do arise, they frequently stem from primary sites elsewhere in the genitourinary tract.^1^^,^^2^ There have been only a handful of documented cases in the surgical literature detailing urethral metastases originating from colorectal cancer.^3^, ^4^, ^5^, ^6^, ^7^, ^8^, ^9^, ^10^, ^11^, ^12^, ^13^, ^14^ Previous case reports highlight management of urethral metastases with options such as surgical resection and chemotherapy with a view to achieve remission. This report provides unique insight into a case in which a palliative approach was adopted with interval imaging. This highlights that each case warrants careful consideration and tailored approaches to diagnosis and treatment.

In Australia, colorectal cancer ranks as the third most commonly diagnosed cancer, and adenocarcinoma represents the predominant histologic subtype, accounting for over 90% of cases.^15^^,^^16^ When colorectal cancers metastasize, they tend to follow a specific pattern, with the liver, lungs, and peritoneum being the most frequent sites of spread.^13^ The infrequency of colorectal cancer metastasizing to the urethra means that appropriate investigations for patients presenting with lower urinary tract symptoms may be overlooked. This rarity underscores the unique challenges that urologists may face when encountering such patients, as there is limited precedent and established guidance for the management of these rare occurrences.

Immunohistochemistry plays a crucial role in differentiating the origin of urethral tumours, especially since there is a morphological overlap between various tumour types. Antibodies targeting epithelium-associated proteins, such as Cytokeratin-7 (CK7) and Cytokeratin-20 (CK20), are often employed in this diagnostic process. CK7 is expressed in carcinomas of the urinary tract but not in colorectal adenocarcinoma, whereas CK20 is present in both urinary and colorectal cancers. A CK7-/CK20+ expression pattern is typical of colorectal adenocarcinoma.^14^^,^^17^ Additionally, CDX2, an intestinal transcription factor expressed in the nuclei of intestinal epithelial cells, aids in identifying metastatic tumours originating from intestinal adenocarcinoma. In the case described here, the combination of adenocarcinoma histology and the pattern of CK7-/CK20+ and CDX2+ expression strongly supported a diagnosis of secondary urethral cancer arising from a known rectal adenocarcinoma primary.

Knowledge of treating secondary urethral tumours of colorectal origin is limited to a few case reports. Treatments described in existing case reports are highly variable and have included surgical or endoscopic resection with or without adjuvant chemotherapy and/or radiotherapy, systemic chemotherapy and/or localised radiotherapy, and palliation. Surgical therapy depends on tumour location and involvement of adjacent structures (such as the corpus cavernosa and bladder) and may include transurethral resection, partial urethrectomy with end-to-end anastomosis, partial or total penectomy, or cystourethrectomy. Palliative treatment can involve a combination of palliative chemotherapy, localised radiotherapy or palliative urinary diversion, often in the form of long-term suprapubic catheterisation.^4^^,^^7^ The variability in the treatment of secondary urethral tumours reflects its rarity and the need for individualised patient-centred treatment after discussion at a multidisciplinary team meeting.

Patients with urethral tumours may present with a variety of symptoms, including haematuria, obstructive voiding symptoms, dysuria, and urinary retention.^7^^,^^9^ This case serves as a reminder that clinicians should maintain a high index of suspicion and a low threshold for investigating new urinary symptoms in individuals with known colorectal cancer. Such vigilance can facilitate early detection and prompt treatment of potential metastatic disease, potentially improving patient outcomes and quality of life.

## CRediT authorship contribution statement

**Faiyaz Rahman:** Writing – original draft. **Shravankrishna Ananthapadmanabhan:** Writing – review & editing. **Jeremy Saad:** Writing – review & editing. **Bishoy Hanna:** Writing – review & editing, Conceptualization. **Isaac Thangasamy:** Writing – review & editing, Supervision. **Celalettin Varol:** Writing – review & editing, Supervision, Conceptualization.

## Statement of ethics

Written informed patient consent has been obtained for publication of this case report and images.

## Funding sources

The authors received no funding for this report.

## Conflicts of interest

All authors have no conflict of interest to declare.
